# The Level of Urine Dipstick Proteinuria and Its Relation to the Risk of Incident Cholelithiasis

**DOI:** 10.2188/jea.JE20190223

**Published:** 2021-01-05

**Authors:** Sung Keun Park, Ju Young Jung, Chang-Mo Oh, Min-Ho Kim, Eunhee Ha, Dong-Young Lee, Jung-Wook Kim, Hee Yong Kang, Jae-Hong Ryoo

**Affiliations:** 1 Total Healthcare Center, Kangbuk Samsung Hospital, Sungkyunkwan University, School of Medicine, Seoul, Korea; 2 Departments of Preventive Medicine, School of Medicine, Kyung Hee University, Seoul, Korea; 3 Ewha Institute of Convergence Medicine, Ewha Womans University Mokdong Hospital, Seoul, Korea; 4 Department of Occupational and Environment Medicine, College of Medicine, Ewha Womans University, Seoul, Korea; 5 Department of Internal Medicine, Veterans Healthcare Service Medical Center, Seoul, Korea; 6 Division of Gastroenterology and Hepatology, Department of Internal Medicine, Kyung Hee University School of Medicine, Seoul, Korea; 7 Department of Anesthesiology and Pain Medicine, Kyung Hee University Hospital, Seoul, Korea; 8 Departments of Occupational and Environmental Medicine, School of Medicine, Kyung Hee University, Seoul, Korea

**Keywords:** urine dipstick test, proteinuria, cholelithiasis

## Abstract

**Background:**

Previous studies have suggested the potential association between renal diseases and gallstone. The extent of proteinuria is recognized as a marker for the severity of chronic kidney disease. However, little data is available to identify the risk of incident gallstone according to the level of proteinuria.

**Methods:**

Using a data of 207,356 Koreans registered in National Health Insurance Database, we evaluated the risk of gallstone according to the levels of urine dipstick proteinuria through an average follow-up of 4.36 years. Study subjects were divided into 3 groups by urine dipstick proteinuria (negative: 0, mild: 1+ and heavy: 2+ or greater). Multivariate Cox-proportional hazard model was used to assess the adjusted hazard ratios (HRs) and 95% confidence intervals (CIs) for incident cholelithiasis according to urine dipstick proteinuria.

**Results:**

The group with higher urine dipstick proteinuria had worse metabolic, renal, and hepatic profiles than those without proteinuria, which were similarly observed in the group with incident cholelithiasis. The heavy proteinuria group had the greatest incidence of cholelithiasis (2.39%), followed by mild (1.54%) and negative proteinuria groups (1.39%). Analysis for multivariate Cox-proportional hazard model indicated that the heavy proteinuria group had higher risk of cholelithiasis than other groups (negative: reference, mild proteinuria: HR 0.97 [95% CI, 0.74–1.26], and heavy proteinuria: HR 1.46 [95% CI, 1.09–1.96]).

**Conclusion:**

Urine dipstick proteinuria of 2+ or greater was significantly associated with increased risk for incident gallstone.

## INTRODUCTION

Gallstone disease is frequently observed in asymptomatic adults.^1^ It is common that gallstone is incidentally discovered on abdominal ultrasonography during health check-up or medical examination for other purposes.^2^^,^^3^ The prevalence of gallstone is 10–15% in adults,^4^ and cholesterol stones constitute 80–90% of gallstones.^1^ Despite the relatively high prevalence, the clinical significance of gallstone tends to be underrated due to no requirement for treatment in most cases of asymptomatic gallstone.^5^ However, specific symptoms like biliary colic can occur in 1–4% of individuals with gallstones each year.^6^ Moreover, observational studies have demonstrated that gallstone is associated with increased cardiovascular morbidity and mortality^7^^,^^8^ independent of features of metabolic syndrome.^9^ Thus, it is clinically meaningful to seek other predisposing conditions for gallstone besides classic risk factors.

Previous studies have provided evidence of the significant association between kidney disease and gallstone disease.^10^^,^^11^ In these studies, presence of renal stone and chronic kidney disease (CKD) were associated with the increased probability of gallstone.^10^^,^^11^ Proteinuria is an important indicator of renal damage as a cardinal symptom of CKD. Urine dipstick test is widely used as an initial screening tool for proteinuria because of simplicity, inexpensiveness, and rapid interpretation of results. The association between kidney disease and gallstone extends to a notion that proteinuria may be a potential indicator in assessing the risk of gallstone disease. However, available data is still insufficient in identifying the association of proteinuria with the risk of gallstone.

Using data from 207,356 subjects registered in Korea National Health Insurance Corporation, we investigated the risk of incident gallstone according to level of proteinuria assessed using urine dipstick test.

## PARTICIPANTS AND METHODS

### Data sources

Our results were obtained from analyzing Korean statistics derived from the Korea National Health Information Database (NHID) operated by Korea National Health Insurance Corporation (NHIC), which provides the National Health Insurance Service (NHIS) to the Korean population. NHIS covers over 97% of the entire population living in South Korea, suggesting that the NHID can represent the medical service usage of the entire Korean population.^12^ Therefore, the NHID is a public database on health care utilization, health screening, and socio-demographic variables for Korean population using NHIS. Most of Korea medical institutions are required to contract with NHIC, providing medical information of their healthcare users and patients to NHIC. Collected medical information is recorded in NHID, and data are open to qualified researchers for the purpose of medical research. To gain access to NHID, researchers should get approval for the subject of research from committee of institutional review board (IRB). After getting approval from IRB, researchers apply for the access to NHID at statistics department affiliated to NHIC. Then, researchers are judged for the safety, ethics, and necessity of research. In cases where researchers are permitted to use NHID, researchers can analyze the statistics of NHID.

Ethics approvals for the study protocol and analysis of the data were obtained from the institutional review board of Kyung Hee University Hospital. The informed consent requirement was exempted by the institutional review board because researchers only accessed retrospectively a de-identified database for analysis purposes.

### Study participants

A total 223,551 participants who received medical health check-up in 2009 included in the National Health Information Database. Of these, we initially excluded 4,039 individuals who had previously had diagnoses for cholelithiasis (International Classification of Disease [ICD] K80) from 2002 to the date before medical health examination in 2009. Among the 219,512 participants, 12,156 participants were excluded based on the following exclusion criteria that might influence cholelithiasis or urine protein: 772 people did not have the information about baseline urine protein in 2009, and 11,404 had previously had the information about the diagnosis of cancer (ICD C00–C97) from 2002 to the date before medical health examination in 2009. Because some participants had more than one exclusion criteria, 207,356 participants were included in the final analysis and were observed for the development of cholelithiasis. When a subject with incident cholelithiasis was identified to die during follow-up, follow-up period was regarded to be from date of health check-up (initial enrollment to study) to date of identified incident cholelithiasis. If subject without incident cholelithiasis died during follow-up, follow-up period was regarded to be from date of initial enrollment to date of death.

The total follow-up period was 904,360 person-years, and average follow-up period was 4.36 (standard deviation [SD], 0.51) years.

### Health survey examinations and laboratory measurements

The general health check-up of NHIC was conducted thorough two stages. The first stage examination is a massive screening test to determine the presence or absence of disease among the general population without symptoms. The second stage examination is consultation for screening test and more detailed examination to confirm the presence of disease. These health examinations also included a questionnaire regarding lifestyle and past medical histories. Study data included physical activity, information provided by a questionnaire, anthropometric measurements, and laboratory measurements. Smoking amount was described as pack-years, which were calculated from the smoking-related questionnaire using the following formula: Number of pack-years = (packs smoked per day) × (years as a smoker). The frequency of alcohol consumption was evaluated, and alcohol intake was defined as at least more than 3 times per week. Physical activity was defined as doing moderate-intensity physical activity at least 30 minutes per day more than 4 days each week or vigorous-intensity physical activity at least 20 minutes per day more than 4 days each week. Body mass index (BMI) was calculated as weight (kg) divided by the square of height (m). Systolic blood pressure (BP) and diastolic BP were measured by trained examiners. The following laboratory data were measured at the same time that these participants underwent health examinations: fasting glucose, total cholesterol, triglyceride, high-density lipoprotein (HDL) cholesterol, low-density lipoprotein (LDL) cholesterol, serum creatinine (SCr), aspartate aminotransferase (AST), alanine aminotransferase (ALT), and γ-glutamyltransferase (GGT). Kidney function was measured with estimated glomerular filtration rate (eGFR), which was calculated using the Chronic Kidney Disease Epidemiology Collaboration equation: eGFR = 141 × min(SCr/*K*, 1)^a^ × max(SCr/*K*, 1)^−1.209^ × 0.993^age^ × 1.018 [if female] × 1.159 [if Black], where SCr is serum creatinine, *K* is 0.7 for females and 0.9 for males, a is −0.329 for females and −0.411 for males, min indicates the minimum of SCr/*K* or 1 and max indicates the maximum of SCr/*K* or 1.^13^

The urine protein level was determined from the results of a single urine dipstick analysis. The results of the urine test were based on a scale that quantified proteinuria as absence, 1+, and 2+ or greater.

### Outcome definitions

The identification of incident cholelithiasis was based on reviewing NHID linked to the department of Statistics Korea in NHIC. Korean medical institutions contracting with NHIC are required to provide the medical information of patients. If gallstone is detected in asymptomatic or symptomatic patients using imaging modalities or surgical operation, medical institutions should register patients with newly identified gallstone into NHID as cholelithiasis with ICD-K80. Our study was based on NHID, so we identified the incidence of cholelithiasis on the basis of ICD-code (ICD-K80) registered in NHID. Reviewing NHID from 2002–2009, we first excluded all of individuals with present ICD-K80, and then enrolled individuals without previously registered ICD-K80 into the study. Of these subjects, those with newly registered ICD-K80 from 2009 through 2013 were identified as cases of incident cholelithiasis.

### Statistical analysis

Study subjects were categorized into three groups using urine dipstick proteinuria as follows: negative (absent proteinuria), mild proteinuria (proteinuria 1+) and heavy proteinuria (proteinuria 2+ or greater).

Data were expressed as means (SD) or medians (interquartile range) for continuous variables and percentages of the number for categorical variables.

The one-way ANOVA and X^2^-test were used to analyze the statistical differences among the characteristics of the study participants at the time of enrollment in relation to the three groups.

The person-years were calculated as the sum of follow-up times from the baseline until the diagnosis time of cholelithiasis development or until the December 31, 2013.

To evaluate the associations between urine dipstick protein levels of three groups and incident cholelithiasis, we used Cox proportional hazards models to estimate adjusted hazard ratios (HRs) and 95% confidence intervals (CIs) for incident cholelithiasis, comparing that of mild proteinuria group and heavy proteinuria group with the negative group. Cox-proportional hazard models were adjusted for multiple confounding factors. In the multivariate models, we included variables that might confound the relationship between the three groups and incident cholelithiasis, which include: age, gender, BMI, systolic BP, fasting glucose, total cholesterol, GGT, eGFR, smoking amount (pack-years), alcohol intake, and physical activity. Subgroup analysis was conducted by gender and age. The median age of study population was 56 years, which was used as a cut-off of age subgroup analysis (group of age ≤55 years and age ≥56 years).

To test the validity of the Cox-proportional hazard models, we checked the proportional hazard assumption. The proportional hazard assumption was assessed using log-minus-log survival function and found to be graphically unviolated. *P* values <0.05 were considered to be statistically significant. All statistical analyses were performed using SAS (version 9.4, SAS Institute, Cary, NC, USA).

## RESULTS

During 904,360 person-years of follow-up, 2,919 (1.41%) incident cases of cholelithiasis developed from 2009 through 2013. Table 1 describes the baseline characteristics of the study participants in relation to three groups of urine protein level. There were significant differences between the three groups in all baseline characteristics except LDL-cholesterol and physical activity. The groups with more proteinuria tended to have worse clinical conditions than those without proteinuria, which was more prominent in the mean values of fasting glucose, triglyceride, eGFR, and SCr than other variables. However, despite the statistical significant difference in *P*-for trend, some variables did not show clinically important differences among groups. In particular, this finding was distinct in the variables related to obesity and liver function, including BMI, AST, ALT, and GGT, which were within normal ranges in all groups.

**Table 1.** Baseline characteristics of participants according to the four groups of urine protein levels

Characteristic	Overall	Urine protein level			

		Negative	Mild	Heavy	*P*-for trend^a^
		(absence, *n* = 201,445)	(1+, *n* = 3,820)	(≥2+, *n* = 2,091)	
Person-years, total	904,360	879,011	16,479	8,869	
Person-years, average	4.36 (0.51)	4.36 (0.50)	4.31 (0.63)	4.24 (0.74)	<0.001
Age, years	57.8 (8.6)	57.7 (8.6)	59.4 (9.2)	59.9 (9.2)	<0.001
Gender					<0.001
Male (%)	117,266 (56.6)	113,680 (56.4)	2,276 (59.6)	1,310 (62.6)	
Female (%)	90,090 (43.4)	87,765 (43.6)	1,544 (40.4)	781 (37.4)	
BMI, kg/m^2^	24.0 (2.9)	24.0 (2.9)	24.5 (3.2)	24.8 (3.4)	<0.001
Systolic BP, mm Hg	125.3 (15.2)	125.1 (15.1)	129.0 (16.8)	131.4 (18.1)	<0.001
Diastolic BP, mm Hg	77.7 (9.9)	77.7 (9.9)	79.7 (10.7)	80.1 (11.3)	<0.001
Total cholesterol, mg/dL	200.4 (37.4)	200.3 (37.3)	202.4 (40.6)	204.9 (45.3)	<0.001
Triglyceride, mg/dL	118 (83–171)	118 (83–170)	131 (90–194)	139 (96–208)	<0.001
HDL-cholesterol, mg/dL	55.4 (32.3)	55.5 (32.5)	53.4 (23.1)	53.0 (25.5)	<0.001
LDL-cholesterol, mg/dL	118.5 (39.1)	118.6 (39.0)	117.6 (40.3)	118.5 (44.7)	0.969
Fasting glucose, mg/dL	100.7 (25.3)	100.3 (24.6)	111.7 (38.1)	118.0 (45.1)	<0.001
SCr, mg/dL	1.15 (1.49)	1.14 (1.46)	1.34 (1.91)	1.50 (2.99)	<0.001
eGFR, mL/min per 1.73 m^2^	80.8 (20.2)	81.1 (20.0)	75.5 (22.7)	70.4 (24.6)	<0.001
AST, U/L	24 (20–29)	24 (20–29)	25 (20–31)	25 (20–33)	<0.001
ALT, U/L	21 (16–29)	21 (16–29)	22 (16–33)	23 (16–34)	<0.001
GGT, U/L	25 (17–41)	25 (17–41)	29 (19–52)	31 (20–58)	<0.001
Smoking amount, pack-years	7.8 (13.8)	7.8 (13.8)	9.3 (15.7)	9.5 (15.9)	<0.001
Alcohol intake, %	14.6	14.5	17.3	17.9	<0.001
Physical activity, %	16.8	16.8	17.0	17.2	0.890
Development of cholelithiasis (%)	2,919 (1.41)	2,810 (1.39)	59 (1.54)	50 (2.39)	<0.001

AST, aspartate aminotransferase; ALT, alanine aminotransferase; BMI, body mass index; BP, blood pressure; eGFR, estimated glomerular filtration rate; GGT, gamma glutamyl transferase; HDL, high-density lipoprotein; LDL, low-density lipoprotein; SCr, serum creatinine.

Data are means (standard deviation), medians (interquartile range), or percentages.

^a^*P*-value by ANOVA-test for continuous variables and Chi square test for categorical variables.

There were 2,919 cases of incident cholelithiasis during follow-up, and the characteristics of these individuals compared with the remainder of cohort are presented in Table 2. In contrast to participants without incident cholelithiasis, those with incident cholelithiasis were older (60.8 [SD, 9.4] vs 57.7 [SD, 8.6] years) and had a less favorable baseline characteristics in BMI, systolic BP, TG, HDL-cholesterol, eGFR, AST, ALT, GGT, and smoking amount. In particular, group with incident cholelithiasis had higher levels in baseline characteristics related to obesity and liver function like BMI, AST, ALT, and GGT. However, all variables did not show the specific direction, and the group without cholelithiasis had higher mean levels in diastolic BP, total cholesterol, LDL-cholesterol, SCr, alcohol intake, and physical activity.

**Table 2.** Comparison between participants with and without incident cholelithiasis

Characteristic	Without incident cholelithiasis	With incident cholelithiasis	*P*-value^a^
	(*N* = 204,437)	(*N* = 2,919)	
Age, years	57.7 (8.6)	60.8 (9.4)	<0.001
Gender			0.076
Male (%)	115,568 (56.5)	1,698 (58.2)	
Female (%)	88,869 (43.5)	1,221 (41.8)	
BMI, kg/m^2^	24.0 (2.9)	24.4 (2.9)	<0.001
Systolic BP, mm Hg	125.3 (15.2)	125.8 (15.4)	0.045
Diastolic BP, mm Hg	77.7 (9.9)	77.6 (9.9)	0.618
Total cholesterol, mg/dL	200.4 (37.4)	197.8 (37.5)	<0.001
Triglyceride, mg/dL	142.1 (94.3)	144.9 (94.1)	0.115
HDL-cholesterol, mg/dL	55.4 (32.2)	54.0 (35.8)	0.035
LDL-cholesterol, mg/dL	118.6 (39.1)	117.0 (38.7)	0.025
Fasting glucose, mg/dL	100.7 (25.3)	102.5 (27.1)	<0.001
SCr, mg/dL	1.15 (1.49)	1.09 (1.25)	0.012
eGFR, mL/min per 1.73 m^2^	80.9 (20.1)	79.5 (19.2)	<0.001
Urine protein, %			<0.001
Absence	97.2	97.3	
1+	1.8	2.0	
≥2+	1.0	1.7	
AST, U/L	26.5 (16.2)	28.3 (20.6)	0.061
ALT, U/L	25.3 (19.0)	27.8 (26.8)	<0.001
GGT, U/L	38.9 (53.3)	49.1 (76.2)	<0.001
Smoking amount, pack-years	7.8 (13.8)	8.8 (15.7)	0.001
Alcohol intake, %	14.6	15.2	0.379
Physical activity, %	16.8	17.6	0.259

AST, aspartate aminotransferase; ALT, alanine aminotransferase; BMI, body mass index; BP, blood pressure; eGFR, estimated glomerular filtration rate; GGT, gamma glutamyl transferase; HDL, high-density lipoprotein; LDL, low-density lipoprotein; SCr, serum creatinine.

Data are expressed as means (standard deviation) or percentages.

^a^*P*-value by *t*-test for continuous variables and Chi square test for categorical variables.

Table 3 shows the HRs and 95% CIs for cholelithiasis according to the three groups. In the unadjusted model, the HRs for cholelithiasis comparing mild and heavy proteinuria group versus the negative group were 1.12 (95% CI, 0.87–1.45) and 1.77 (95% CI, 1.33–2.34), respectively (*P* for trend <0.001). Adjustment for covariates attenuated this association, but statistical significance was maintained in the heavy proteinuria group (HR 1.46; 95% CI, 1.09–1.96). After adjusting for covariates, cholelithiasis was significantly associated with BMI, age, alcohol intake, smoking, and GGT.

**Table 3.** Hazard ratios for the incidence of cholelithiasis according to the three groups of urine protein level

	Person-year	Incidence cases	Incidence rate (per 10,000 person-years)	HR (95% CI)^a^	

				Unadjusted	Adjusted model
**Urine protein level**					
negative	879,012	2,810	31.9	1.00 (reference)	1.00 (reference)
mild	16,479	59	35.8	1.12 (0.87–1.45)	0.97 (0.74 –1.26)
heavy	8,869	50	56.4	1.77 (1.33–2.34)	1.46 (1.09–1.96)
*P* for trend				<0.001	0.005
Age					1.04 (1.03–1.05)
Gender (female vs male)					0.95 (0.87–1.04)
BMI					1.055 (1.042–1.068)
Systolic BP					0.995 (0.992–0.997)
Fasting glucose					1.001 (0.999–1.002)
Total cholesterol					0.998 (0.997–0.999)
GGT					1.002 (1.002–1.003)
eGFR					1.001 (0.999–1.003)
Smoking amount, pack-years					1.004 (1.001–1.006)
Alcohol intake					1.125 (1.003–1.262)
Physical activity					0.967 (0.877–1.066)

BMI, body mass index; BP, blood pressure; CI, confidence interval; eGFR, estimated glomerular filtration rate; GGT, gamma glutamyl transferase; HR, hazard ratio.

^a^Multivariate adjusted model was adjusted for age, gender, BMI, systolic BP, fasting glucose, total cholesterol, GGT, eGFR, smoking amount (pack-year), alcohol intake, and physical activity.

Negative: urine dipstick proteinuria 0, mild: urine dipstick proteinuria 1+, heavy: urine dipstick proteinuria ≥2+.

Gender subgroup analysis indicated that heavy proteinuria in women was significantly associated with increased risk of incident cholelithiasis (HR 1.68; 95% CI, 1.06–2.65) even after adjusting for covariates (eTable 1). Men also showed the significant association in the unadjusted model (HR 1.65; 95% CI, 1.15–2.37), which disappeared after adjustment for covariates (HR 1.31; 95% CI, 0.89–1.92). In age subgroup analysis (eTable 2), group of age ≥56 years showed a significant association between heavy proteinuria and incident cholelithiasis (HR 1.44; 95% CI, 1.01–2.03), but the group of age ≤55 years did not show a significant association after adjustment for covariates (HR 1.47; 95% CI, 0.85–2.55).

## DISCUSSION

In a longitudinal analysis of nationwide data, we evaluated the risk of incident cholelithiasis according to the levels of urine dipstick proteinuria. Our result indicated that urine dipstick proteinuria of 2+ or greater was significantly associated with increased risk of cholelithiasis. The analysis for baseline characteristics of study subjects provides a potential mechanism for this finding. The subjects with higher urine dipstick proteinuria tended to have worse metabolic and renal conditions, which were similarly observed in subjects with incident cholelithiasis. These findings suggest that unfavorable clinical conditions had a role in the development of gallstone. This inference is supported by the previous studies displaying the role of metabolic derangements like insulin resistance, obesity, and dyslipidemia on the development of gallstone, proteinuria, and CKD.^14^^–^^16^ Thus, it is speculated that the metabolic milieu contributing to proteinuria triggers the pathophysiological processes involved in the development of gallstone. However, it is interesting that our results were statistically significant even after adjusting for covariates, including conventional risk factors for gallstone like age, gender, BMI, systolic BP, fasting glucose, total cholesterol, GGT, alcohol intake, and physical activity. This result indicates that proteinuria may be an independent risk factor for gallstone. Previous studies have also demonstrated that renal diseases related to proteinuria are potentially associated with gallstone. In a cross-sectional study of 2,686 men and 2,087 women in Taiwan,^11^ the prevalence of gallstone was 13.1% in the group of patients with CKD, and 4.9% in the group of patients without CKD (*P* < 0.001). Additionally, it has been demonstrated that the prevalence of gallstone was significantly higher in patients with end-stage renal disease (ESRD) treated with dialysis compared with a non-uremic group.^17^^,^^18^ Observational studies have shown the significant association between gallstone and renal stone.^19^^,^^20^ These results give rise to a hypothesis that considerable overlap may exist between pathophysiological mechanisms of renal diseases and gallstone disease. Moreover, considering that proteinuria is a clinical manifestation of renal diseases, including CKD and renal stone, these results may link proteinuria to gallstone. However, previous studies are limited in presenting the direct influence of proteinuria on incident gallstone. Their limitations are attributable to cross-sectional design,^10^^,^^11^ less generalizability of results derived only from ESRD patients,^17^^,^^18^ and weak causative relationship between renal stone and proteinuria.^19^^,^^20^ Furthermore, several studies have reported that the prevalence of gallstone did not differ between dialysis patients and healthy controls.^21^^–^^23^ In contrast, we analyzed the longitudinal relationship between the level of urine dipstick proteinuria and the risk of incident gallstone, which may be an advantage in identifying the clinical implication of renal disease related to proteinuria as a risk factor for gallstone.

In our analysis, heavy proteinuria (≥2+) was significantly associated with the increased risk of gallstone, whereas mild proteinuria (1+) did not show a statistically significant association with gallstone. Previous studies have demonstrated that the level of proteinuria was a reliable baseline factor deeply correlated with the rate of eGFR decline and progressive CKD.^24^^,^^25^ Thus, it is postulated that the heavy proteinuria group had a higher proportion of advanced CKD with uremia than the mild proteinuria group over the follow-up period. A uremic state can derange the complex process of neural and hormonal factors controlling gallbladder motility.^26^^–^^28^ The neural and hormonal imbalance may alter gallbladder motility, promoting gallstone formation via the stasis of gallbladder in CKD patients.^26^^–^^28^ However, we cannot guarantee that uremic state induced by CKD is a major mechanism for the association between proteinuria and cholelthiasis in our study. We could not evaluate the variation of renal function during follow-up due to not performing follow-up measurements of SCr and eGFR. Further studies should investigate the long-term association among baseline proteinuria, variation of renal function, and risk of cholelithiasis.

The merits of the study are the robust number of study subjects, well-organized medical records (including diagnosis of cholelithiasis), and laboratory measurements based on credible nationwide data. These advantages enable us to quantify the risk of incident cholelithiasis according to the levels of urine dipstick proteinuria.

Nonetheless, we acknowledge the limitation of the study. First, the level of proteinuria was evaluated only using urine dipstick test. Although the urine dipstick test is widely available in screening proteinuria, it is insufficient to precisely quantify proteinuria. Second, the follow-up period of 4.36 years on average was relatively short. The cumulative incidence of cholelithiasis was 2.5% in our study, but longer follow-up might lead to both lower incidence rate and higher cumulative incidence for cholelithiasis. Third, our study was conducted only for relatively elderly Koreans with a mean age of 57.8 (SD, 8.6) years. Our study showed that the prevalence of +1 proteinuria and ≥2+ proteinuria is 1.8% and 1.0%, respectively. However, in a cohort study of 18,201,275 Koreans with mean age of 45.3 (SD, 14.6) years based on NHID, prevalence of 1+ proteinuria and ≥2+ proteinuria was 1.18% (*n* = 214,883) and 0.56% (*n* = 103,745), respectively.^29^ The higher prevalence of proteinuria in our study may be attributable to the older age of our subjects. Fourth, we could not verify validity on the incidence of cholelithiasis in the study due to lack of validation on the incidence of cholelithiasis from previous analysis through NHID. Fifth, despite the possibility of loss to follow-up during follow-up, we could not conduct sensitivity analysis due to the limitation of our raw data. NHID was not designed for research, but rather for investigation of health status of Koreans. Therefore, we could not identify the information needed for sensitivity analysis.

These limitations warrant the necessity of further studies with more precise modalities quantifying proteinuria, longer follow-up, and large number of subjects, including younger age groups.

In conclusion, individuals with more proteinuria had higher incidence of cholelithiasis, and urine dipstick proteinuria of 2+ or greater was significantly associated with increased risk of cholelithiasis. These results add to the evidence for a hypothesis that the presence of renal disease reflected by proteinuria is an independent risk factor for gallstone disease.

## APPENDIX A. SUPPLEMENTARY DATA

eTable 1. Hazard ratios for the incidence of cholelithiasis according to the three groups of urine protein level in gender subgroups (men and women)

eTable 2. Hazard ratios for the incidence of cholelithiasis according to the three groups of urine protein level in age subgroups of ≤55 years and ≥56 years
