# Supplementary material for: The Level of Urine Dipstick Proteinuria and Its Relation to the Risk of Incident Cholelithiasis
Source: J Epidemiol. 2021 Jan 5;31(1):59–64. doi: 10.2188/jea.JE20190223 (PMC7738639; doi:10.2188/jea.JE20190223)
Supplement: Supplementary file 1 [file je-31-059-s001.pdf]

**eTable 1.** Hazard ratios for the incidence of cholelithiasis according to the three groups of urine protein level in gender subgroups (men and women)

| | HR (95% CI)[a] | | | |
|---|---|---|---|---|
| | Men (n=117,266) | | Women (n=90,090) | |
| | Unadjusted | Adjusted model | Unadjusted | Adjusted model |
| **Urine protein level** | | | | |
| negative | 1.00 (reference) | 1.00 (reference) | 1.00 (reference) | 1.00 (reference) |
| mild | 1.02 (0.76–1.45) | 0.85 (0.59 –1.22) | 1.26 (0.86–1.86) | 1.13 (0.76 –1.69) |
| heavy | 1.65 (1.15–2.37) | 1.31 (0.89–1.92) | 1.95 (1.25–3.04) | 1.68 (1.06–2.65) |
| P for trend | 0.010 | 0.175 | 0.003 | 0.018 |
| Age | | 1.05 (1.04–1.06) | | 1.03 (1.02–1.04) |
| BMI | | 1.067 (1.049–1.086) | | 1.045 (1.026–1.064) |
| Systolic BP | | 0.993 (0.990–0.997) | | 0.997 (0.993–1.001) |
| Fasting blood glucose | | 1.001 (0.999–1.003) | | 1.000 (0.997–1.002) |
| Total cholesterol | | 0.998 (0.997–0.999) | | 0.999 (0.998–1.001) |
| GGT | | 1.002 (1.002–1.003) | | 1.004 (1.003–1.004) |

| | | |
|---|---|---|
| eGFR | 1.002 (0.999–1.004) | 1.000 (0.997–1.003) |
| Smoking amount, pack-years | 1.003 (1.000–1.006) | 1.014 (0.998–1.031) |
| Alcohol intake | 1.084 (0.962–1.222) | 1.591 (0.996–2.541) |
| Physical activity | 0.973 (0.860–1.101) | 0.988 (0.841–1.160) |

BMI, body mass index; BP, blood pressure; CI, confidence interval; eGFR, estimated glomerular filtration rate; GGT, gamma glutamyl transferase; HR, hazard ratio.

[a]Multivariate adjusted model was adjusted for age, BMI, systolic BP, fasting blood glucose, total cholesterol, GGT, eGFR, smoking amount (pack-years), alcohol intake, and physical activity.

Negative: urine dipstick proteinura 0, mild: urine dipstick proteinura 1+, heavy: urine dipstick proteinuria ≥2+

**eTable 2.** Hazard ratios for the incidence of cholelithiasis according to the three groups of urine protein level in age subgroups of ≤55 years and ≥56 years

| | HR (95% CI)[a] | | | |
|---|---|---|---|---|
| | Age ≤55 years (n=101,428) | | Age ≥56 years (n=105,928) | |
| | Unadjusted | Adjusted model | Unadjusted | Adjusted model |
| **Urine protein level** | | | | |
| negative | 1.00 (reference) | 1.00 (reference) | 1.00 (reference) | 1.00 (reference) |
| mild | 1.00 (0.61–1.64) | 0.99 (0.61 –1.63) | 1.11 (0.82–1.50) | 0.95 (0.69 –1.30) |
| heavy | 1.70 (1.00–2.87) | 1.47 (0.85–2.55) | 1.68 (1.21–2.34) | 1.44 (1.01–2.03) |
| P for trend | 0.056 | 0.142 | 0.003 | 0.046 |
| Age | | 1.06 (1.03–1.09) | | 1.04 (1.03–1.04) |
| Gender (female vs. male) | | 1.09 (0.93–1.27) | | 0.88 (0.79–0.98) |
| BMI | | 1.070 (1.047–1.094) | | 1.049 (1.033–1.065) |
| Systolic BP | | 0.991 (0.987–1.003) | | 0.996 (0.993–0.999) |
| Fasting blood glucose | | 1.001 (0.998–1.003) | | 1.001 (0.999–1.002) |

| | | |
|---|---|---|
| Total cholesterol | 0.999 (0.997–1.001) | 0.998 (0.997–1.000) |
| GGT | 1.002 (1.001–1.003) | 1.003 (1.002–1.003) |
| eGFR | 1.002 (0.999–1.006) | 1.000 (0.997–1.003) |
| Smoking amount, pack-years | 1.005 (0.999–1.010) | 1.003 (1.000–1.006) |
| Alcohol intake | 1.090 (0.903–1.316) | 1.153 (0.996–1.333) |
| Physical activity | 1.072 (0.898–1.280) | 0.930 (0.827–1.047) |

BMI, body mass index; BP, blood pressure; CI, confidence interval; eGFR, estimated glomerular filtration rate; GGT, gamma glutamyl transferase; HR, hazard ratio.

[a]Multivariate adjusted model was adjusted for age, gender, BMI, systolic BP, fasting blood glucose, total cholesterol, GGT, eGFR, smoking amount (pack-years), alcohol intake, and physical activity.

Negative: urine dipstick proteinura 0, mild: urine dipstick proteinura 1+, heavy: urine dipstick proteinuria ≥2+
